# The relationship between uncertainty in illness and caregiving capacity in primary caregivers of patients with first-time ischemic stroke: the roles of psychological adaptation and perceived social support

**DOI:** 10.3389/fpsyg.2026.1707967

**Published:** 2026-08-03

**Authors:** Guanrong Guo, Xuemei Li, Huan Wang, Yingdong Lin, Chen Li, Huili Zhu, Dongxiang Zheng

**Affiliations:** 1 School of Nursing, Jinan University, Guangzhou, Guangdong, China; 2 Department of Neurology, The First Affiliated Hospital of Jinan University, Guangzhou, China

**Keywords:** caregiving capacity, first-time ischemic stroke, mediating effect, perceived social support, primary caregivers, psychological adaptation, uncertainty in illness

## Abstract

**Background:**

In China, family members typically assume primary caregiving responsibilities for stroke patients after treatment. However, the onset of a first ischemic stroke often occurs abruptly, and caregivers, typically spouses, adult children, or other non-professionals, may assume this responsibility without psychological preparation or caregiving experience. This situation may be associated with higher uncertainty in illness. In turn, caregiving confidence may be reduced, potentially compromising caregiving capacity. Therefore, factors associated with primary caregivers’ caregiving capacity should be examined to inform supportive strategies and reduce caregiver burden.

**Method:**

Standardized scales were used to assess primary caregivers' uncertainty in illness, psychological adaptation, perceived social support, and caregiving capacity. Correlations were analyzed, and mediation and moderation effects were tested.

**Result:**

Psychological adaptation (*r* = 0.708, *p* < 0.01) and perceived social support (*r* = 0.675, *p* < 0.01) were both positively correlated with caregiving capacity. Psychological adaptation partially mediated the association between uncertainty in illness and caregiving capacity, accounting for 29.98% of the total effect. Perceived social support significantly moderated the association between uncertainty in illness and psychological adaptation (*β* = −0.01, *p* < 0.001). In the low perceived social support group, uncertainty in illness showed a stronger negative association with psychological adaptation, whereas this association was attenuated in the high perceived social support group.

**Conclusion:**

Uncertainty in illness was negatively associated with caregiving capacity both directly and indirectly through psychological adaptation. Perceived social support may mitigate the adverse association between uncertainty in illness and psychological adaptation. Interventions aimed at reducing uncertainty in illness, enhancing psychological adaptation, and strengthening social support may help improve caregiving capacity among primary caregivers of patients with first-time ischemic stroke.

## 1 Introduction

Stroke is among the leading causes of death and disability in adults (Tu et al., 2023). Ischemic stroke is the most common type, accounting for approximately 80% of all stroke cases (Saini et al., 2021). Acute management includes undergoing thrombolytic therapy, endovascular procedures, and other interventions aimed at improving clinical outcomes. However, approximately 70% of stroke survivors continue to experience functional impairments, including limb weakness, language difficulties, cognitive deficits, and psychological disorders, which substantially limit their activities of daily living (Miao et al., 2024). Consequently, post-stroke rehabilitation often relies heavily on primary caregivers, who assume primary responsibility for patient care and rehabilitation during hospitalization and after discharge at home (Choi et al., 2018). Unlike other progressive chronic conditions, the sudden onset of a first-time ischemic stroke often leaves primary caregivers unprepared, requiring caregiving responsibilities to be assumed abruptly. It has been reported that up to 90% of stroke patients receive care primarily from family members who are untrained and insufficiently prepared (Xu et al., 2025).

Uncertainty in illness is defined as a cognitive state that arises when insufficient information is available to adequately interpret or categorize an illness and is characterized by tension and ambiguity (Mishel, 1988). Research has shown that primary caregivers of stroke patients must contend with the acute stroke event, an unpredictable prognosis, and the need to coordinate rehabilitation services (Ponzini et al., 2022). For primary caregivers of patients who have experienced a first-time ischemic stroke, concerns commonly include the risk of recurrence, management of sequelae, and financial burden. These challenges are frequently accompanied by a strong sense of insecurity among primary caregivers. This psychological condition may be associated with impaired decision-making and quality of care, and may be related to family dysfunction (Byun et al., 2016).

Mishel’s Revised Uncertainty in Illness Theory (RUIT) provides a theoretical framework for understanding how uncertainty is experienced and managed during illness. This theory comprises four components: predisposing factors, assessment, management, and outcome (Zhang, 2017). Predisposed factors are considered key determinants of uncertainty in illness and include three elements: the stimulus framework, the cognitive ability, and the helpers. Social support, as a component of the “helpers” domain, may reduce the uncertainty of illness directly or indirectly. Uncertainty in illness is conceptualized as a neutral element of the illness experience; its impact depends on individual appraisal, and coping strategies are shaped by these appraisals. For primary caregivers of patients with first-time ischemic stroke, the need to manage a complex condition and an unpredictable prognosis is likely to generate substantial uncertainty. As a crucial psychosocial resource, social support may enhance caregivers' understanding of illness-related information, strengthen cognitive appraisal, and promote positive psychological adaptation, and may reduce the negative consequences associated with uncertainty (Ong et al., 2018). From this perspective, social support may facilitate reappraisal of the uncertainty of illness as a manageable challenge and may also influence coping strategies and psychological adaptation, which in turn affect caregiving capacity.

Although the relationship between uncertainty in illness and caregiving capacity has been examined, the underlying mechanisms have not been fully elucidated. According to the RUIT (Zhang, 2017), primary caregivers of patients with first-time ischemic stroke may experience heightened uncertainty due to the sudden onset, an uncertain prognosis, and the risk of recurrence. This cognitive status may directly influence caregiving ability. It has been reported that uncertainty in illness may be associated with reduced physical caregiving skills and lower situational adaptation among caregivers, along with increased anxiety and lower overall caregiving capacity. Psychological adaptation may play a crucial role in understanding this process. Caregivers who fail to effectively integrate information about the illness or lack cognitive restructuring skills may be more likely to appraise uncertainty as a “threat” rather than an “opportunity,” resulting in poor psychological adaptation (Zhang, 2017; Zhou and Zhou, 2024). This maladaptation may be reflected in maladaptive coping strategies and reduced self-efficacy, which may further exacerbate perceived caregiver burden and inadequacy. Collectively, these findings suggest that uncertainty in illness may affect caregiver capacity through psychological adaptation as a mediating pathway.

Perceived social support is defined as the extent to which individuals perceive care and assistance from family members, friends, and others in their social network (Fan et al., 2018). Perceived social support is considered a key coping resource when individuals are exposed to stress (Bareket-Bojmel et al., 2021). According to RUIT, social support functions as a structural resource that may reshape the stimulus framework by providing disease-management information, emotional support, and financial assistance, thereby buffering the adverse effects of uncertainty. A study by Ryan et al. (2003) in patients with cancer reported that higher social support was associated with lower perceived insecurity, anxiety, and depression. Levels of uncertainty in illness have been shown to be associated with social support, coping strategies, and other factors (Han et al., 2021). Therefore, perceived social support may moderate the association between primary caregivers' uncertainty in illness and psychological adaptation in patients with first-time ischemic stroke.

In summary, this study aims to investigate the impact of primary caregivers' uncertainty in illness on caregiving capacity. The relationships among uncertainty in illness, perceived social support, psychological adaptation, and caregiving capacity will be examined, and the indirect pathways linking uncertainty in illness to caregiving capacity will be tested. This research is expected to provide a theoretical basis for health professionals to develop targeted interventions. Based on the above theories and findings, the following hypotheses are proposed:

*H1*: Uncertainty in illness is negatively associated with primary caregivers’ caregiving capacity and psychological adaptation.

*H2*: Psychological adaptation mediates the association between uncertainty in illness and the caregiving capacity of primary caregivers.

*H3*: Perceived social support moderates the association between uncertainty in illness and psychological adaptation.

**Figure 1:**
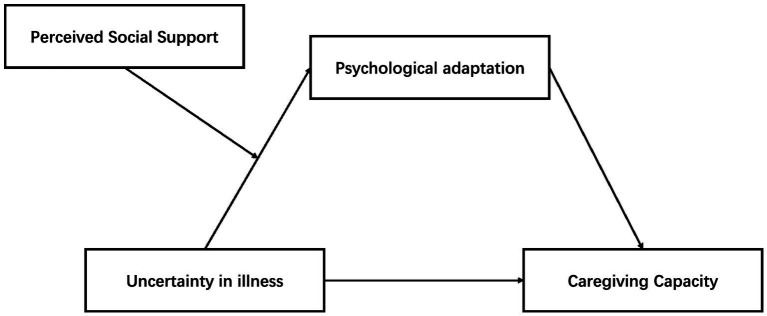
Conceptual model diagram. This model illustrates the hypothesized mediating role of psychological adaptation between uncertainty in illness and caregiving capacity. Perceived social support serves as a moderating variable within the conceptual framework. Arrows indicate proposed directional pathways.

The conceptual model is shown in Figure 1.

## 2 Method

A cross-sectional study was conducted from May 2023 to January 2024, and 200 primary caregivers of patients with a first-ever ischemic stroke were recruited using convenience sampling at a hospital in Guangzhou, Guangdong, China.

Sample size was estimated using Gorsuch's rule of thumb (Gorsuch, 1983), which recommends that the sample size should be 5 to 10 times the number of predictor variables. To ensure sufficient statistical power under the most stringent conditions, we adopted a worst-case scenario approach, assuming that all 14 background variables collected in this study (4 patient characteristics and 10 caregiver characteristics) might be included as covariates in the regression models. Combined with the core theoretical model variables, including the independent variable (X), moderator (W), interaction term (X × W), and mediator (M), the equation predicting psychological adaptation contained a maximum of 17 predictor variables (X ± W ± X × W ± 14 covariates). Using the most conservative estimate (5 times the number of predictors), the minimum required sample was 17 × 5 = 85; accounting for a 20% invalid response rate, the adjusted minimum was 102. The maximum estimate (10 times the number of predictors) was 17 × 10 × (1 ± 20%) = 204. The target sample size range was 102 to 204 participants. The final sample of 200 valid cases fell within this range, indicating adequate statistical power.

Patient inclusion criteria were as follows: first-time occurrence of ischemic stroke meeting the diagnostic criteria of the Chinese Guidelines for the Diagnosis and Treatment of Acute Ischemic Stroke (2023) (Chinese Medical Association Neurology Branch, 2024); and ischemic stroke confirmed by CT or MRI. Patients were excluded if they were admitted to the intensive care unit (ICU) or had severe cardiac, hepatic, or renal impairment, or malignant tumors.

Inclusion criteria for primary caregivers were as follows: ① a spouse, adult child, or other family member providing unpaid care to the patient. ② Responsible for the majority of the caregiving tasks and providing at least 4 h of care per day. If multiple caregivers were available, the individual providing the greatest amount of care and assuming primary caregiving responsibilities was selected. ③ Conscious with normal cognitive and language functions. ④ Willing to participate and able to provide written informed consent.

Exclusion criteria were as follows: ① individuals unable to complete the questionnaire despite the investigator's assistance and clarification. ② Caregivers who are paid employees, such as domestic helpers or nursing assistants.

## 3 Ethical review

This study was approved by the Ethics Committee of the First Affiliated Hospital of the University of Jinan (JUNUKY-2023-0010). Before data collection, authorization was obtained from the nursing department and department heads. The study objectives and procedures were explained to primary caregivers before the questionnaires were administered. Patients and their primary caregivers were informed of their right to withdraw at any time without affecting their access to standard medical care. Participants were also assured that the survey was anonymous, that the data would be used solely for research purposes, and that personal information would not be disclosed to third parties.

## 4 Measure

### 4.1 Demographic information

Background information was collected on the demographic characteristics of patients with first-time ischemic stroke and their caregivers. Patient characteristics included age, type of health insurance, length of hospital stay, and treatment type. Caregiver characteristics included age, gender, marital status, educational level, relationship with the patient, monthly household disposable income, place of residence, presence of chronic illness, physical health status, and whether a co-caregiver was available.

### 4.2 Mishel uncertainty in illness scale for family member (MUIS-FM)

The Parents’ Perception Uncertainty Scale-Family (PPUS-FM), developed by Mishel (1983), is a self-report measure designed to assess family members’ illness-related uncertainty following a patient’s diagnosis. In 2010, Chinese scholar Cui (2010) translated the scale into Chinese and evaluated its reliability and validity. The content validity index was 0.87. Following the pilot study, the overall Cronbach's *α* for the 30-item scale was 0.89. The scale comprises four dimensions: ambiguity, complexity, information deficiency, and unpredictability. Responses are rated on a 5-point Likert scale from 1 (strongly disagree) to 5 (strongly agree), and items 6, 9, 11, 19, 23, 25, 27, 28, 29, and 30 are reverse-scored. Higher scores indicate greater uncertainty in illness.

### 4.3 Psychological adaptation scale (PAS)

Biesecker et al. (2013) developed the Psychological Adaptation Scale (PAS), which was designed to assess psychological adaptation in individuals with chronic illness and their caregivers. Mengjia et al. (2021) translated, culturally adapted, and validated the scale in China. The Chinese version of the PAS comprises four dimensions: coping, self-esteem, social skills, and psychological development, with 20 items in total. The total scale demonstrated excellent internal consistency (Cronbach'sα = 0.900). Items are rated on a 5-point Likert scale, yielding a total score ranging from 20 to 100. Higher scores indicate better psychological adaptation among caregivers.

### 4.4 Perceived social support scale (PSSS)

The Perceived Social Support Scale (PSSS), developed by Zimet et al. (1990), is a self-report instrument designed to measure individuals’ perceived multidimensional social support. The scale was subsequently translated into Chinese by Qianjin (2001). The scale comprises three dimensions: family support, friend support, and other support. Items are rated on a 7-point Likert scale, with scores ranging from 12 to 36 indicating low support levels, 37 to 60 indicating moderate support levels, and 61 to 84 indicating high support levels.

### 4.5 The family caregiver task inventory (FCTI)

The Family Caregiving Task Inventory (FCTI) was developed in 1983 by Clark and Rakowski (1983) and was translated and culturally adapted into Chinese in 2011 by Lee and Mok (2011). The inventory comprises 5 dimensions: Learning to cope with a new role, Providing care according to the care-receiver’s needs, Managing one's own emotional needs, Appraising supportive resources, and Balancing caregiving needs and one’s own needs. Items are rated on a 3-point scale, with higher scores indicating lower caregiving competence. For statistical analyses in the present study, scores were reverse-coded so that higher values reflected greater caregiving competence.

### 4.6 Statistical analysis

IBM SPSS Statistics 26.0 was used for data management and statistical analyses, including factor analysis, correlation analysis, reliability and validity testing, and assessment of common method bias. Categorical variables were summarized as frequencies and percentages. Continuous variables with a normal distribution were presented as mean ± standard deviation (SD), whereas non-normally distributed variables were presented as median (interquartile range, IQR). To examine factors associated with the outcomes, independent-samples t tests, one-way analysis of variance (ANOVA), rank-sum tests, and multiple linear regression were performed. Pearson correlation analyses were conducted to assess associations among uncertainty in illness of primary caregivers, psychological adaptation, perceived social support, and caregiver capacity. Stepwise multiple linear regression was used to identify factors associated with these variables. It is important to note that, given the cross-sectional nature of the study design, all regression coefficients and indirect pathways estimated using the PROCESS macro represent statistical associations rather than causal effects. The terms 'mediation' and 'moderation' are used in the statistical sense to describe the decomposition of variance and conditional relationships, consistent with established methodological conventions (Hayes, 2013). To examine the proposed moderated mediation model, the SPSS PROCESS macro was used. Model 4 was first estimated as a nested baseline mediation model to assess whether psychological adaptation mediated the association between uncertainty in illness and caregiving capacity. The primary hypothesis was tested using Model 7, which evaluated whether perceived social support moderates the first stage of the mediation process (the path from uncertainty in illness to psychological adaptation). Model 7 simultaneously estimated the mediation and moderation components within a single integrated framework and produced conditional indirect effects at different levels of the moderator. All continuous variables were mean-centered prior to analysis to facilitate interpretation of the interaction term. Bootstrap resampling (5,000 samples) was used to generate 95% confidence intervals for the conditional indirect effects.

## 5 Result

### 5.1 Common method bias test

Using exploratory factor analysis, the variance explained by the first unrotated factor extracted from the full scale was 31.316%, which was below the 40% threshold. This does not indicate any significant bias in the common method in this study, which allows for a subsequent statistical analysis.

### 5.2 General Information of the patient and primary caregivers

A total of 201 patients with first-time ischemic stroke and their primary caregivers were surveyed; 200 questionnaires were valid, yielding a response rate of 99.50%. As shown in Table 1, Univariate analyses identified statistically significant differences in primary caregivers' caregiving capacity by patient length of hospital stay and treatment modality, as well as primary caregiver age, gender, education attainment, monthly household disposable income, and physical health status (*p* < 0.05).

**Table 1:** Univariate analysis of general characteristics and caregiving capacity among primary caregivers of patients with first-time stroke (*n* = 200).

Characteristics		*n* (%)	Caregiving capacity (mean ± SD)	F/t	*p*
Patient demographic information					
Age (years)	18–45	12 (6)	36.25 ± 11.22	0.25	0.780
	46–59	66 (33)	37.83 ± 10.39		
	≥60	122 (61)	38.39 ± 8.14		
Medical expense payment methods	Medical insurance	164 (82)	38.19 ± 9.15	0.36	0.719
	Out-of-pocket	36 (18)	37.58 ± 8.99		
Hospitalization duration(days)	1–7	29 (14.5)	43.21 ± 6.01	6.14	0.001
	8–14	139 (69.5)	38.04 ± 9.07		
	15–30	27 (13.5)	33.37 ± 9.43		
	≥30	5 (2.5)	34.80 ± 9.78		
Treatment methods	Thrombolytic therapy	6 (3)	40.17 ± 8.23	3.14	0.046
	Endovascular therapy	75 (37.5)	36.05 ± 8.82		
	Medical therapy	118 (59)	39.28 ± 9.15		
Primary caregivers general information					
Age (years)	18–45	84 (42)	40.20 ± 8.20	6.99	0.001
	46–59	83 (41.5)	37.77 ± 8.92		
	≥60	33 (16.5)	33.45 ± 10.12		
Gender	Male	86 (43)	39.58 ± 8.55	2.043	0.042
	Female	114 (57)	36.95 ± 9.37		
Marital status	Married	181 (90.5)	39.58 ± 9.14	−1.314	0.190
	Single, widowed, divorced, or separated	19 (9.5)	36.95 ± 8.48		
Educational attainment	Elementary school and below	21 (10.5)	29.52 ± 8.18	11.762	0.000
	Junior high school	61 (30.5)	36.90 ± 9.06		
	High school and technical secondary school	65 (32.5)	38.65 ± 8.25		
	College and above	53 (26.5)	42.13 ± 8.02		
The relationship with patients	Spouse	74 (37)	36.39 ± 10.29	3.337	0.069
	Children	113 (56.5)	39.60 ± 7.47		
	Siblings	4 (2)	25.75 ± 11.3		
	Parents	6 (3)	34.50 ± 11.73		
	Others	3 (1.5)	46.00 ± 5.29		
Monthly Per Capita Disposable Income of Households (RMB)	≤1000	8 (4)	30.750 ± 11.00	12.459	0.000
	1001–3000	48 (24)	33.60 ± 9.18		
	3001–5000	68 (34)	37.60 ± 8.60		
	>5001	76 (38)	42.10 ± 7.38		
Residence	Urban	133 (66.5)	38.95 ± 8.87	1.912	0.057
	Rural	67 (33.5)	36.36 ± 9.36		
Suffering from chronic diseases	Yes	46 (23)	38.95 ± 10.02	−3.75	0.000
	No	154 (77)	36.36 ± 8.43		
Physical health status	Good	105 (52.5)	40.77 ± 8.20	19.127	0.000
	Average	87 (43.5)	36.13 ± 8.69		
	Poor	8 (4)	24.00 ± 6.63		
Have co-caregivers	Yes	105 (52.5)	39.80 ± 7.58	2.819	0.005
	No	95 (47.5)	36.18 ± 10.24		

### 5.3 Scale scores and correlation analysis

As shown in Table 2, the mean score for uncertainty in illness was 83.07 ± 13.49. The mean scores for caregiving capacity were 38.08 ± 9.10, psychological adaptation were 60.25 ± 18.45, and perceived social support were 61.27 ± 13.66. Caregiving Capacity was negatively correlated with uncertainty in illness (*r* = −0.758, *p* < 0.01), positively correlated with perceived social support (*r* = 0.675, *p* < 0.01), and psychological adaptation (*r* = 0.708, *p* < 0.01). Psychological adaptation was negatively correlated with uncertainty in illness (*r* = −0.692, *p* < 0.01) and positively correlated with perceived social support (*r* = 0.709, *p* < 0.01). Perceived social support was negatively correlated with uncertainty in illness (*r* = −0.617, *p* < 0.01).

**Table 2:** Correlation analysis of primary caregivers’ uncertainty in illness, psychological adaptation, perceived social support, and caregiving capacity (*n* = 200).

Variable	*M* ± SD	1	2	3	4
Uncertainty in illness	83.07 ± 13.49	1			
Caregiving capacity	38.08 ± 9.10	−0.758**	1		
Psychological adaptation	60.25 ± 18.45	−0.692**	0.708**	1	
Perceived social support	61.27 ± 13.66	−0.617**	0.675**	0.709**	1

***p < 0.001.

### 5.4 Analysis of the mediating role of psychological adaptation

In the PROCESS mediation and moderation analyses, covariates were selected based on empirical evidence. Specifically, all variables that showed significant associations with caregiving capacity in univariate analyses (Table 1) were entered simultaneously into a multiple linear regression model (Table 3). Among these, only monthly per capita disposable income of households remained a significant independent predictor of caregiving capacity (*p* = 0.043), in addition to the core study variables. Therefore, monthly per capita disposable income of households was included as a covariate in the PROCESS models to control for its potential confounding effect.

**Table 3:** Mediating effect of psychological adaptation between primary caregivers' uncertainty in illness and caregiving capacity (*n*=200).

Variable	Caregiving capacity (Model 1)			Psychological adaptation (Model 2)			Caregiving capacity (Model 3)		
	*β*	*t*	95%CI	*β*	*t*	95%CI	*β*	*t*	95%CI
Uncertainty in illness	−0.48	−14.39	−0.54, −0.41	−0.89	−11.75	−1.03, −0.73	−0.33	−8.28	−0.41, −0.25
Psychological adaptation							0.16	5.54	0.10, 0.22
*R* ^2^	0.59			0.50			0.65		
Δ*R*^2^	/			/			0.06		
*F*	141.76**			96.78**			118.96**		

**p < 0.001, Δ*R*^2^ = Model 3's *R*^2^ minus Model 1's *R*^2^, representing the additional variance explained after including the psychological adaptation variable. All models controlled for monthly per capita disposable income of households.

The PROCESS Model 4 was first estimated as a nested baseline mediation model, with uncertainty in illness as the independent variable, caregiving capacity as the dependent variable, and psychological adaptation as the mediator. Monthly per capita disposable income of households was included as a covariate in this model. As shown in Table 3, in Model 1, uncertainty in illness significantly predicted lower caregiving capacity [*β* = −0.48, *t* = −14.39, *p* < 0.001, 95% CI (−0.54, −0.41)]. In Model 2, uncertainty in illness significantly predicted lower psychological adaptation [*β* = −0.89, *t* = −11.75, *p* < 0.001, 95% CI (−1.03, −0.73)]; In Model 3, after psychological adaptation was included, the direct effect of uncertainty in illness decreased [*β* = −0.33, *t* = −8.28, *p* < 0.001, 95% CI (−0.41, −0.25)]. Psychological adaptation was positively associated with caregiving capacity [*β* = 0.16, *t* = −5.54, *p* < 0.001, 95% CI (0.10, 0.22)], indicating that psychological adaptation partially mediates the association between uncertainty in illness and caregiving capacity. Adding psychological adaptation significantly increased the explained variance in caregiving capacity (Δ*R*^2^ = 0.06). The indirect effect was evaluated using 5,000 bootstrap resamples with a 95% confidence interval. As shown in Table 4, the total effect of uncertainty in illness on caregiving capacity was −0.477, comprising a direct effect of −0.334 and an indirect effect via psychological adaptation of −0.143. The indirect effect via psychological adaptation accounted for 29.98% of the total effect. The effect size of this indirect effect was f^2^ = 0.17, corresponding to a medium effect according to Cohen's criteria. Taken together, these results supported Hypothesis 1.

**Table 4:** Decomposition table for testing the mediating effect of psychological adaptation between primary caregivers' uncertainty in illness and caregiving capacity (*n* = 200).

Effect	*β*	SE	*t*	*p*	Bootstrap (95%CI)	Relative effect size	Effect size ( $f^{2}$ )
Total effect	−0.477	0.033	−14.389	0.000	(−0.542, −0.411)	100%	
Direct effect	−0.334	0.040	−8.282	0.000	(−0.413, −0.254)	70.02%	
Indirect effect	−0.143	0.036	/	/	(−0.223, −0.081)	29.98%	0.17

The effect size $f^{2}$ for the indirect effect was calculated as $f^{2}=\Delta R^{2}/(1-R_{\text{full model}}^{2})$ , where $\Delta R^{2}=.06$ and $R_{\text{full model}}^{2}=.65$ . According to Cohen’s (1988) guidelines, $f^{2}=0.02$ , 0.15, and 0.35 represent small, medium, and large effect sizes, respectively. All models controlled for monthly per capita disposable income of households.

### 5.5 The moderating role of perceived social support

PROCESS Model 7 as the final integrated conditional process model, in which perceived social support (W) moderated the X → M path while the mediation and direct paths were estimated within the same model. Monthly per capita disposable income of households was included as a covariate in this model. The interaction between uncertainty in illness and perceived social support significantly predicted lower psychological adaptation (*β* = −0.01, *p* < 0.001). Accordingly, as shown in Table 5, the moderating effect of perceived social support was statistically significant.

**Table 5:** Analysis of the moderating effect of perceived social support on the relationship between primary caregivers' uncertainty in illness and caregiving capacity.

Variable	Psychological adaptation			Caregiving capacity		
	*β*	*t*	95%CI	*β*	*t*	95%CI
Uncertainty in illness (X)	−0.49	−6.10***	−0.66, −0.33	−0.33	−8.28***	−0.41, −0.25
Perceived social support (W)	0.67	8.22***	0.51, 0.83			
XW	−0.01	−2.76**	−0.02, −0.01			
Psychological adaptation (M)				0.16	5.54***	0.10, 0.22
*R* ^2^	0.63			0.65		
*F*	81.41***			118.96***		

***p < 0.001. All models controlled for monthly per capita disposable income of households.

### 5.6 Simple slope analysis

**Figure 2:**
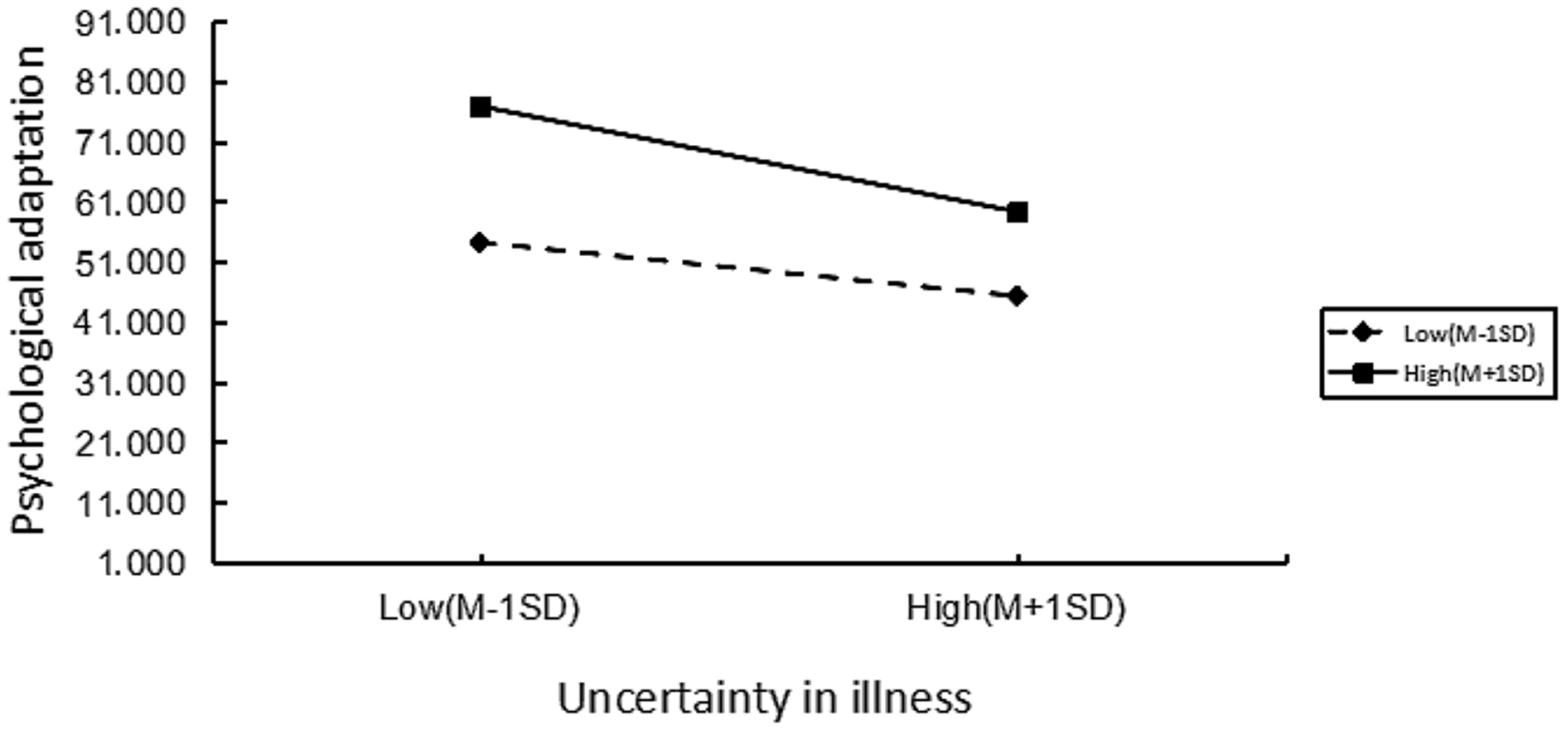
Simple slope diagram of the moderating effect of perceived social support. Simple slope analysis shows the relationship between uncertainty in illness and psychological adaptation at high (+1 SD) and low (−1 SD) levels of perceived social support. The negative effect of uncertainty on adaptation was weaker among caregivers reporting high social support.

To clarify whether the association between uncertainty in illness and psychological adaptation varied by perceived social support, participants were categorized into high and low perceived social support groups using a mean ± 1 SD split. Figure 2 illustrates the moderating effect of perceived social support on the association between uncertainty in illness and psychological adaptation. In the low perceived social support group, uncertainty in illness was significantly associated with lower psychological adaptation. In the high-perceived social support group, this negative association was attenuated. Compared with the low perceived social support group, the indirect effect of illness-related uncertainty on caregiving capacity through psychological adaptation was reduced in the high perceived social support group, as shown in Figure 2.

## 6 Discussion

A negative association was observed between primary caregivers’ uncertainty in illness and caregiving capacity, indicating that higher uncertainty in illness was associated with lower caregiving capacity. Psychological adaptation partially mediated the association between uncertainty in illness and caregiving capacity, and perceived social support moderated this indirect effect.

### 6.1 Main interpretation

A moderated mediation model was tested to examine the association between uncertainty in illness and caregiving capacity. The study was organized into three components. First, Hypothesis 1 was supported: uncertainty in illness was negatively correlated with caregiving capacity and psychological adaptation. This finding is consistent with the theoretical proposition that uncertainty disrupts individuals’ existing cognitive frameworks. When uncertainty cannot be clearly interpreted by primary caregivers, higher cognitive load may be observed, which may be associated with lower caregiving capacity. This pattern is consistent with the findings reported by Ponzini et al. (2022). In addition, the positive association between psychological adaptation and caregiving capacity aligns with the proposition that “adaptation is the outcome of coping with uncertainty.” When uncertainty is integrated into coping processes through psychological adaptation, growth-oriented adaptation may be achieved. Such adaptation may be reflected in enhanced coping capacity, which may, in turn, be associated with improved caregiving capacity, consistent with findings reported by Yan and Solina (2024).

Second, Hypothesis 2 was supported, indicating that psychological adaptation mediates the relationship between uncertainty in illness and caregiving capacity. This uncertainty reduces caregiving capacity by weakening psychological adaptation. Although previous studies have examined the association between primary caregivers' uncertainty in illness and caregiving capacity (Mei et al., 2023; Jing and Dongmei, 2021), research on the mediating role of psychological adaptation in this relationship remains relatively limited. Psychological adaptation refers to an individual's capacity for cognitive and behavioral adjustments under disease stress. The level of this adaptation depends on how disease events are evaluated and the ability to integrate coping resources (Mishel, 1983). Objectively, uncertainty may be related to increased cognitive load in processing disease information. Subjectively, it may be related to cognitive confusion, making it difficult for caregivers to integrate disease experiences into an orderly cognitive framework, which may correlate with reduced psychological adaptation. The sudden onset of first-time ischemic stroke and the complexity of the rehabilitation process may be associated with a series of role conflicts and emotional reactions in caregivers, which may correlate with reduced caregiving efficacy (Yan and Solina, 2024). Furthermore, poor psychological adaptation is associated with difficulties in caregiving-related behaviors, such as difficulties in role transition, failure in emotional management, and inadequate resource utilization, which may correlate with lower caregiving capacity (Butz et al., 2012). Collectively, our study suggests that uncertainty in illness may be related to poorer psychological adaptation among caregivers of patients with first-time acute ischemic stroke, which may correlate with lower caregiving capacity.

Third, Hypothesis 3 was supported: perceived social support moderated the indirect association between uncertainty in illness and caregiving capacity. Perceived social support may function as a crucial external coping resource, attenuating the indirect pathway linking uncertainty in illness to caregiving capacity. Specifically, higher perceived social support was associated with better psychological adaptation and, in turn, greater caregiving capacity (Mishel, 1983). Caregivers with low perceived social support may have fewer external resources to manage the cognitive demands of illness, which may be associated with greater vulnerability to poorer psychological adaptation when confronting uncertainty (Xu et al., 2024). Under such conditions, poor psychological adaptation may be associated with diminished caregiving capacity, thereby reducing overall caregiving capacity. Despite the importance of social support, prior studies have reported that many caregivers of stroke survivors have unmet social support needs (Noorian et al., 2025). These findings suggest that social support systems for this population may require strengthening. Accordingly, interventions designed to enhance social support for primary caregivers of patients with ischemic stroke may be warranted (Wanjun et al., 2024).

Finally, scores for primary caregivers’ uncertainty in illness, psychological adaptation, and perceived social support were examined. Uncertainty in illness and psychological adaptation were at moderate levels, consistent with findings reported by Wang et al. (2022), Li et al. (2024), and Yan and Solina (2024). Perceived social support was at a moderate-to-high level and exceeded levels reported among primary caregivers of patients with chronic diseases in Cui (2010). The family support subscale showed the highest score. Among caregivers, 56.5% were adult children and 37% were spouses, suggesting close kinship ties and strong caregiving obligations. Within traditional Chinese family culture, a sudden serious illness may activate a “shared-burden” mechanism among immediate family members. This mechanism may provide sustained financial assistance, emotional companionship, and practical caregiving support, thereby contributing to higher family support scores. A study by Adarve and Osorio (2020) reported that support from family members was associated with improved ability to manage disease-related problems, whereas emotional support from friends and relatives may help reduce uncertainty. Furthermore, a supportive social network may enhance treatment adherence and facilitate psychological regulation, which may reduce uncertainty and improve primary caregivers’ caregiving capacity (Tan et al., 2021; Li et al., 2019).

### 6.2 Practical and theoretical implications

From a theoretical perspective, new empirical support was provided for Mishel’s Uncertainty in Illness Theory. First, the theory’s core proposition was supported: the impact of uncertainty depends on individuals’ appraisal and available coping resources. Psychological adaptation, as a mediator, may reflect caregivers’ appraisal of uncertainty, whereas perceived social support, as a moderator, may indicate how external resources reshape cognitive frameworks.

From a practical perspective, the findings have important implications for clinical intervention. First, uncertainty in illness among primary caregivers may be reduced through the timely provision of information and guidance. Information support may help primary caregivers develop realistic expectations regarding the illness and reduce cognitive confusion. Second, caregivers’ psychological adaptation should be assessed, and psychological support strategies, such as cognitive behavioral therapy and mindfulness-based interventions, may be introduced to facilitate role transition. Third, caregivers’ social support networks should be strengthened, particularly by enhancing family involvement and support. The transition from hospital to home may represent a particularly psychologically stressful period for caregivers. Comprehensive intervention programs implemented during this period may improve caregivers’ caregiving capacity.

### 6.3 Clinical implications

Based on the study findings, primary caregivers should be provided with clear, accessible information regarding stroke prognosis, the rehabilitation process, and complication prevention during hospitalization. This may be accomplished through one-on-one bedside education and distribution of standardized care manuals. After discharge, ongoing guidance may be delivered via telephone follow-up or mobile health application notifications to help caregivers develop realistic expectations. For caregivers with lower psychological adaptation, individualized counseling may be provided to facilitate reappraisal of uncertainty in illness as manageable. During the transition from hospital to home, home visits may be conducted by community nurses or follow-up staff to assess support needs and link families to community resources, such as day care centers and nursing homes, thereby promoting continuity of support.

## 7 Conclusion

This study examined associations among primary caregiver uncertainty in illness, psychological adaptation, perceived social support, and caregiver capacity in patients with first-time ischemic stroke. In the hypothesized model, psychological adaptation was specified as a mediator of the association between uncertainty in illness and caregiver capacity. Perceived social support was found to moderate this indirect pathway, such that higher perceived social support attenuated the negative association between uncertainty in illness and psychological adaptation. Accordingly, interventions targeting these factors may improve the caregiver capacity of primary caregivers. Future research should further examine the temporal dynamics of these variables and identify effective strategies to strengthen perceived social and psychological support. Potential clinical approaches include interventions targeting psychological adaptation and uncertainty in illness, such as online platforms for psychological support, advocacy for policies that expand social support, and structured training programs for primary caregivers. These approaches are intended to mitigate the adverse effects of uncertainty in illness on caregivers’ ability to provide effective care. In addition, examining how these associations vary across cultural contexts, family structures, and socioeconomic conditions could inform the development of more targeted interventions.

### 7.1 Limitations and future directions

A cross-sectional design was used, which captures associations among variables at a single time point and does not permit inference regarding causal relationships or temporal dynamics. Associations among uncertainty in illness, psychological adaptation, and caregiving capacity may be bidirectional. In the future, longitudinal designs could be adopted, with data collected at multiple time points to test causal pathways and explore gender or relationship-type differences in the moderated mediation model. Second, the sample was drawn from a single tertiary hospital in Guangdong Province, which may limit generalizability. Future studies should expand the sampling frame, conduct multicenter research, and assess whether the moderated mediation model differs by caregiver gender and caregiver–patient relationship type. Such variation could influence the model’s applicability and the tailoring of intervention strategies. Although the model was informed by Mishel’s Uncertainty in Illness Theory, other factors, such as caregiver health status and patient functional impairment may also contribute. Future research may integrate additional theoretical perspectives to develop more comprehensive explanatory models.

## Data Availability

The raw data supporting the conclusions of this article will be made available by the authors, without undue reservation.
